# Incidence and Associated Risk Factors for Lactic Acidosis Induced by Linezolid Therapy in a Case–Control Study in Patients Older Than 85 Years

**DOI:** 10.3389/fmed.2021.604680

**Published:** 2021-02-25

**Authors:** Tingting Liu, Chao Hu, Jionghe Wu, Miao Liu, Yifan Que, Jiang Wang, Xiangqun Fang, Guogang Xu, Hongxia Li

**Affiliations:** ^1^Department of Pulmonary and Critical Care Medicine, The Second Medical Center, National Clinical Research Center for Geriatric Diseases, Chinese People's Liberation Army General Hospital, Beijing, China; ^2^The Second Medical Center, National Clinical Research Center for Geriatric Diseases, Chinese People's Liberation Army General Hospital, Beijing, China; ^3^Second Medical Centre, Institute of Gerontology, Chinese People's Liberation Army General Hospital, Beijing, China; ^4^Centre of Pulmonary and Critical Care Medicine, Chinese People's Liberation Army General Hospital, Beijing, China

**Keywords:** risk prediction model, risk factors, linezolid-induced lactic acidosis, very elderly, serum lactic acid

## Abstract

**Background:** Serum lactic acid is considered a prognostic indicator in critically ill patients. However, studies on linezolid-induced lactic acidosis (LILA) are still limited. Individuals older than 85 years old (very elderly) have limited capacity for organ compensation, and LILA data from these patients are lacking. In this study, we evaluated the risk factors for LILA in patients older than 85 years and established a risk prediction model for geriatric practice.

**Methods:** In this retrospective cohort study, blood gas analysis data and arterial lactate levels were monitored in patients older than 85 years during the use of teicoplanin or linezolid. After propensity score matching analyses, we compared the incidence of lactic acidosis between the teicoplanin and linezolid therapy groups and identified the risk factors of LILA.

**Results:** The incidence of lactic acidosis was found to be much lower in the group receiving teicoplanin than those receiving linezolid therapy (0 vs. 35.7%; *p* < 0.0001). A duration of linezolid therapy ≥ 9 days [odds ratio (OR), 3.541; 95% confidence interval (CI), 1.161–10.793; *p* = 0.026], an arterial blood glucose level ≥ 8 mmol/L (OR, 4.548; 95% CI, 1.507–13.725; *p* = 0.007), and a high sequential organ failure assessment score (OR, 1.429; 95% CI, 1.213–1.685; *p* < 0.0001) were risk factors for LILA. The constructed risk model could be used to predict LILA (area under the curve, 0.849; specificity, 65.1%; sensitivity, 91.4%, with a negative predictive value of 93.2% and a positive predictive value of 59.3%).

**Conclusions:** LILA can occur in patients older than 85 years after a relatively shorter duration of linezolid therapy. Therefore, close monitoring of blood gas and arterial lactate levels during linezolid therapy in the very elderly population is necessary.

## Introduction

Serum lactic acid is produced by anaerobic glycolysis, mainly in the skeletal muscles, skin, erythrocytes, and central nervous system (1). Clinically, elevated lactate levels often represent hypoxia in tissues, so lactate is commonly used to evaluate tissue perfusion and prognosis in critically ill patients (2, 3). It has been reported that when the blood lactate level is higher than 10 mmol/L, the mortality rate is >80% (4, 5).

Elevated lactate levels caused by drugs do not necessarily indicate hypoxia, and such high lactate levels gradually decrease back to the normal range after drug withdrawal. Among different types of drug-induced lactic acidosis, little is known about linezolid-induced lactic acidosis (LILA). Linezolid is the first clinically available oxazolidinone antibacterial agent against infections caused by multidrug-resistant gram-positive pathogens (6–8). The most common adverse reaction to linezolid is reversible myelosuppression (anemia, thrombocytopenia, and leukopenia) (9), and some rare adverse reactions are toxic optic neuropathy (10, 11), irreversible peripheral neuropathy (12–14), and lactic acidosis (15, 16). The incidence of LILA has been reported to be between 5 and 33%, affecting the survival of patients (17–20).

However, large-sample studies on the risk factors for LILA and relevant data on the very elderly population are less reported so far. Hence, we analyzed the risk factors for LILA and established a risk prediction model.

## Methods

### Study Design and Participants

This retrospective cohort case–control study was conducted at the Second Medical Centre of Chinese People's Liberation Army General Hospital.

Blood gas analysis and arterial lactate levels of patients older than 85 years were monitored during teicoplanin or linezolid treatment from October 2016 to April 2019 in our hospital. To compare lactic acidosis incidence between patients receiving teicoplanin and linezolid therapy, the baseline characteristics of patients in those groups were adjusted using propensity score matching. Patients receiving linezolid therapy were divided into the lactic acidosis and non-lactic acidosis groups, and the risk factors for LILA were evaluated.

Patients with shock, patients with respiratory failure (partial pressure of oxygen ≤ 60 mmHg) or liver failure (Child–Pugh classification C), patients with heart failure (NYHA classification 3 or higher), and those using drugs that affect lactate levels (such as metformin, salicylates, and nucleotide reverse transcriptase inhibitors) or receiving renal replacement therapy were excluded.

Patients older than 85 years had a low compensatory ability, with lactic acidosis defined as a serum pH <7.35 and arterial lactate level ≥ 3.5 mmol/L in the study. End-point referred to the time when the medication was stopped. The baseline and end-point lactate levels were obtained from blood gas analyses performed when teicoplanin or linezolid therapy was started and stopped. An arterial blood collection syringe (BD Preset, UK) was used to collect 1 ml of radial artery blood, which will be sent for testing within 15 min. A blood gas analyzer (Roche Cobas B221, Switzerland) was used to analyze arterial blood pH and lactate levels. The World Health Organization-The Uppsala Monitoring Centre Method was used to evaluate LILA, and the causal relationship was certain or probable/likely to be classified as LILA. Linezolid was from Pfizer (USA) with two different formulations: linezolid injection (0.6 g: 300 ml) and linezolid tablet (0.6 g). The dosage regimen was administrated as linezolid injection (0.6 g qm) and linezolid tablet (0.6 g qn) for all patients.

### Data Collection

Baselines of the following clinical and laboratory variables were collected retrospectively from the electronic medical records system: sex, age, duration of linezolid therapy, infection site, the use of invasive ventilation, comorbid diseases such as chronic obstructive pulmonary disease, pulmonary fibrosis, coronary heart disease, hypertension, atrial fibrillation, diabetes mellitus), chronic kidney disease (CKD), neurological disease, malignant tumor, and thyroid hypofunction; laboratory indexes including the levels of serum creatinine, albumin, hemoglobin, creatine kinase, lactate dehydrogenase, alanine dehydrogenase, aspartate transaminase, troponin I (TNI), pro-brain natriuretic peptide, D-dimer, arterial blood glucose and estimated glomerular filtration rate (eGFR), sequential organ failure assessment (SOFA) score, and arterial lactate levels at baseline and the end-point. SOFA score was assessed when starting teicoplanin or linezolid treatment.

### Statistical Analysis

Quantitative data with normal distributions were expressed in the form of means and standard deviations and were analyzed by *t*-tests. Data with non-normal distributions were conveyed as medians and interquartile ranges and were assessed with the Mann–Whitney U test. Moreover, categorical variables were described as frequencies, with comparisons being made by using the chi-square test. To adjust for the significant differences in patients' baseline characteristics in the two treatment groups, we performed propensity score matching by implementing the nearest-neighbor matching in a 1:1 ratio. Factors with significant differences in univariate analysis were entered into a multivariate binary logistic regression model (forward: LR) to determine their independent effects. The results of the binary logistic regression model are presented as odds ratios (ORs) and the associated 95% confidence intervals (CIs). The sensitivity and specificity of the risk prediction model were tested using the receiver operating characteristic curve analysis. The 30-day mortality rates of patients in the lactic acidosis and non-lactic acidosis groups were assessed by the Kaplan–Meier method with the Breslow test. Variables with *p*-values < 0.05 were considered statistically significant. All analyses were performed using IBM SPSS statistical software package version 23.0 (SPSS, Chicago, IL, USA).

## Results

### Patient Characteristics and Clinical Factors

**Figure 1:**
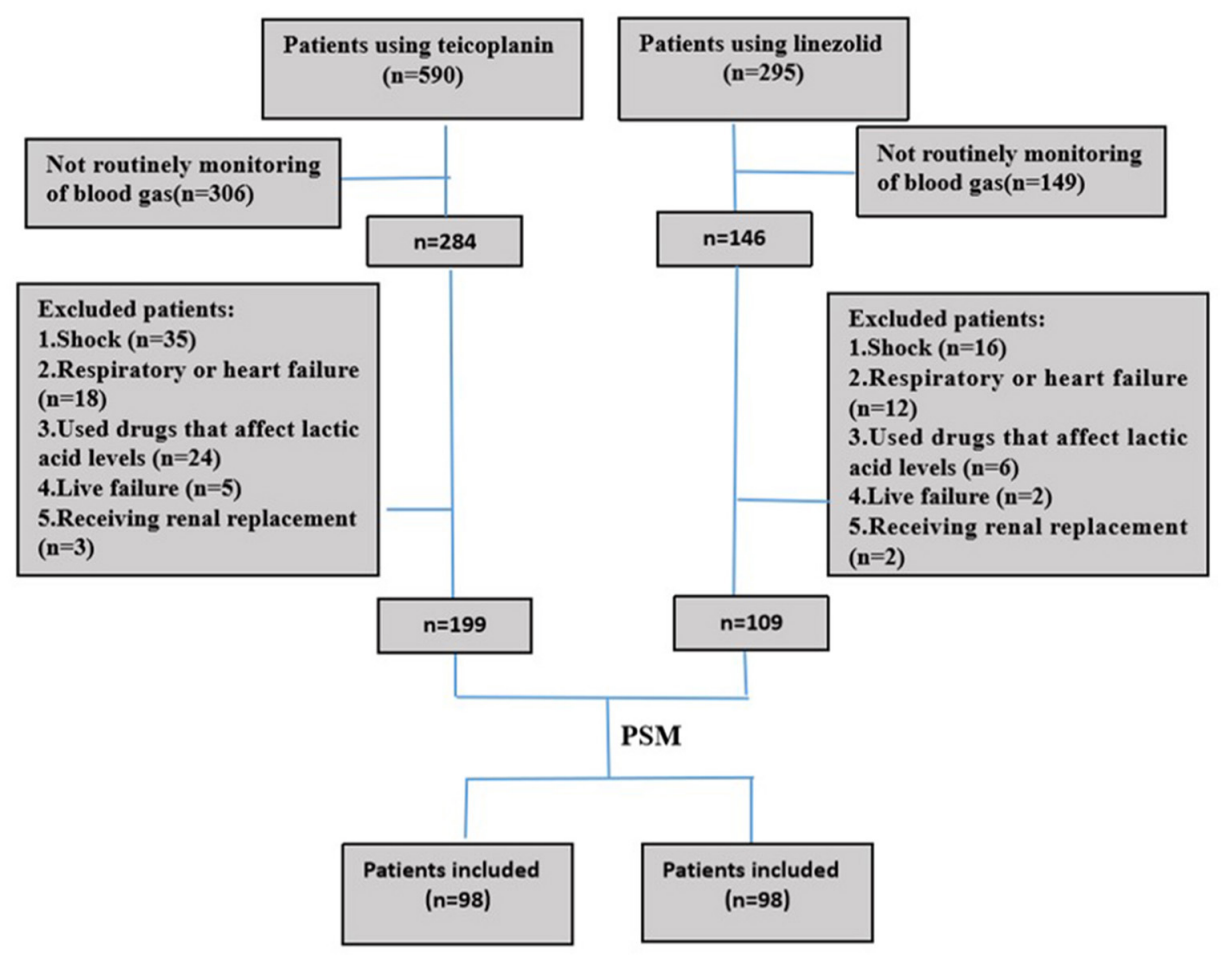
Screening of patients using teicoplanin or linezolid during the study period.

In this retrospective cohort study, 199 and 108 patients were administered teicoplanin or linezolid therapy, respectively (Figure 1). Of the patients, 15.3% (15/98) were skin and soft tissue infections, and most were single streptococcal infections. Pulmonary infections were found in 84.7% (83/98) of the patients, mainly hospital-acquired pneumonia or ventilator-associated pneumonia. They were mixed infections based on gram-negative bacteria (*Acinetobacter baumannii, Klebsiella pneumoniae*, or *Pseudomonas aeruginosa*). All patients received the standard dose of antibiotics. Patient characteristics and clinical factors such as infection site, underlying disease (coronary heart disease, atrial fibrillation, and neurological disease), and SOFA scores are shown in Supplementary Material. Propensity score matching was used to adjust for significant differences in the baseline characteristics of the two groups. As a result, 98 patients were matched in each of the teicoplanin and linezolid therapy groups (Figure 1). The balance in baseline characteristics between the two groups improved considerably (Table 1).

**Table 1:** Patient demographic and clinical characteristics after propensity score matching (nearest neighbor matching).

	**Teicoplanin (*n* = 98)**	**Linezolid (*n* = 98)**	***P*-value**
Male sex, *N* (%)	88 (89.8%)	89 (90.8%)	0.809^*^
Age, years, median (IQR)	94 (91, 96.25)	94 (91, 97)	0.739^#^
Duration of linezolid, days, median (IQR)	8.5 (6.75, 12.00)	9 (6, 11.25)	0.937^#^
Infection sites, *N* (%)			0.400^*^
Pulmonary infection	87 (88.8%)	83 (84.7%)	
Non-pulmonary infection	11 (11.2%)	15 (15.3%)	
Invasive ventilation, *N* (%)	38 (38.8%)	34 (34.7%)	0.553^*^
Underlying disease			
COPD, *N* (%)	80 (81.6%)	78 (79.6%)	0.718^*^
Pulmonary fibrosis, *N* (%)	13 (13.3%)	11 (11.2%)	0.663^*^
Coronary heart disease, *N* (%)			0.817^*^
Stable	88 (89.8%)	87 (88.8%)	
Coronary ischemia	10 (10.2%)	11 (11.2%)	
Hypertension, *N* (%)	79 (80.6%)	78 (79.6%)	0.858^*^
Atrial fibrillation, *N* (%)	53 (54.1%)	44 (44.9%)	0.199^*^
Diabetes mellitus, *N* (%)	45 (45.9%)	43 (43.9%)	0.774^*^
Chronic kidney disease, *N* (%)	46 (46.9%)	49 (50.0%)	0.668^*^
Neurological disease, *N* (%)	30 (30.6%)	29 (29.6%)	0.876^*^
Malignant tumor, *N* (%)	11 (11.2%)	12 (12.2%)	0.824^*^
Thyroid hypofunction, *N* (%)	3 (3.1%)	5 (5.1%)	0.718^*^
Serum creatinine, mg/dl, median (IQR)	124.0 (78.0, 160.0)	110.5 (72.75, 160.0)	0.257^#^
SOFA, median (IQR)	9 (6, 13)	9 (6, 12)	0.233^#^
Lactic acidosis, *N* (%)	0	35 (35.7%)	<0.0001^*^

*IQR, interquartile range; COPD, chronic obstructive pulmonary disease; SOFA, sequential organ failure assessment*.

* *Chi-square test*.

# *Mann–Whitney U test*.

### Arterial Lactate Levels at Baseline and the End-Point in Patients in the Teicoplanin and Linezolid Therapy Groups

After propensity score matching, the incidence rates of lactic acidosis in the teicoplanin and linezolid therapy groups were 0% (0/98) vs. 35.7% (35/98), respectively, with significant differences (*p* < 0.0001, Table 2). No significant difference was found in the baseline of arterial lactate levels between the two groups. In contrast, the arterial lactate level at the end-point was significantly higher in the linezolid therapy group than that in the teicoplanin therapy group (*p* < 0.0001, Table 2).

**Table 2:** Arterial lactate at baseline and the end-point of patients.

	**Teicoplanin (*n* = 98)**	**Linezolid (*n* = 98)**	***P*-value**
**Arterial lactate, (mmol/L)**			
At baseline	1.2 (0.9, 1.525)	1.2 (0.9, 1.4)	0.450^#^
At end-point	1.1 (0.9, 1.425)	2.6 (1.875, 3.7)	<0.0001^#^
*P* value	0.312^#^	<0.0001^#^	
Lactic acidosis, *N* (%)	0	35 (35.7%)	<0.0001^#^

# *Mann–Whitney U test*.

### Univariate Analysis of Risk Factors for Linezolid-Induced Lactic Acidosis

**Figure 2:**
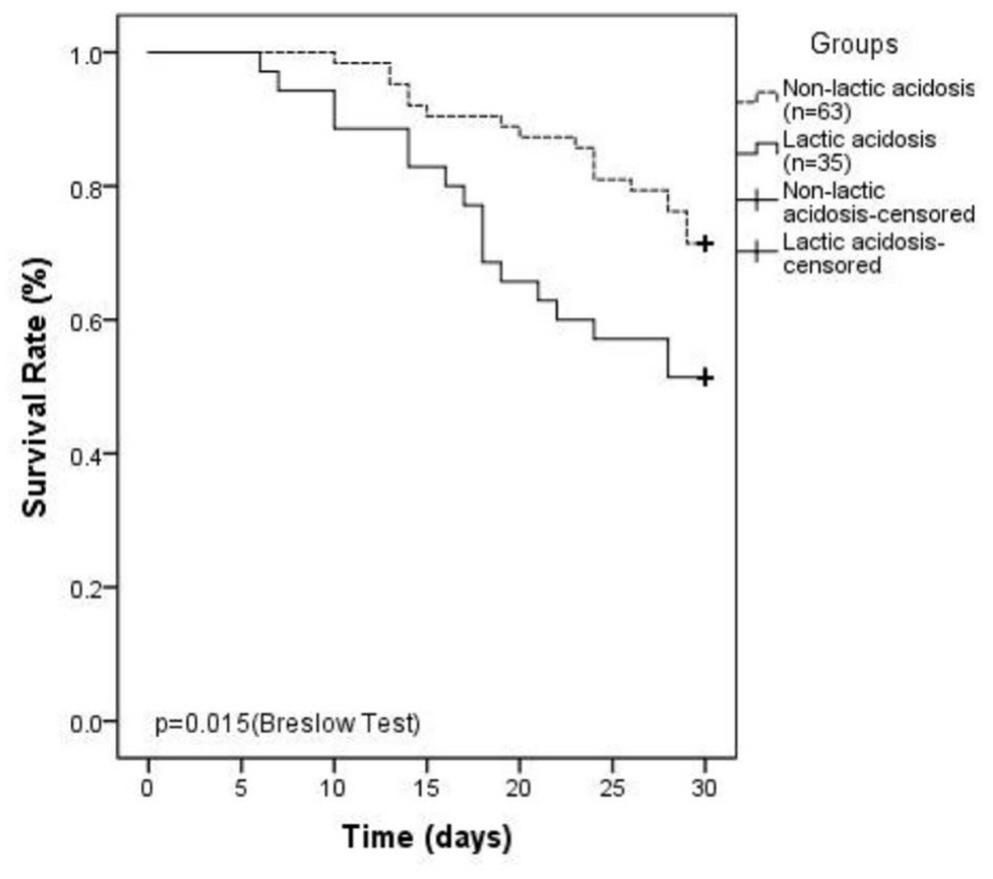
Kaplan–Meier plot showing 30-day survival ratesinlinezolid-induced lactic acidosis group is lower than that in non-lactic acidosis group (48.6 vs. 28.6%, *P* = 0.015).

In the linezolid therapy group, there were 35 patients with lactic acidosis and 63 patients with non-lactic acidosis. The median duration of linezolid therapy was 10 (7, 12) days in the lactic acidosis group and 8 (5, 11) days in the non-lactic acidosis group (*p* = 0.053, Table 3). The numbers of patients with CKD in the lactic acidosis and non-lactic acidosis groups were 24 (68.6%) and 31 (49.2%) (*p* = 0.064, Table 3), respectively. Arterial lactate levels at the end-point were significantly different between the two groups [4.6 (3.7, 5.5) mmol/L vs. 2.1 (1.6, 2.6) mmol/L, *p* < 0.0001, Table 3]. The clinical parameters, such as serum creatinine, hemoglobin, TNI, D-dimer, arterial blood glucose levels, and eGFR were significantly different between the two groups (*p* < 0.05, Table 3). The SOFA scores of the two groups were 10 (9, 15) and 6 (5, 9) (*p* < 0.0001, Table 3), respectively. The 30-day mortality rates were 48.6 and 28.6%, respectively, and were significantly different (*p* = 0.015, Table 3, Figure 2).

**Table 3:** Univariate analysis of risk factors of linezolid-induced lactic acidosis.

	**Lactic acidosis (*n* = 35)**	**Non-lactic acidosis (*n* = 63)**	***P*-value**
Male sex, *N* (%)	32 (91.4%)	57 (90.5%)	1.000^*^
Age, years, median (IQR)	94 (90, 96)	94 (91, 97)	0.471^#^
Duration of linezolid, days, median (IQR)	10 (7, 12)	8 (5, 11)	0.053^#^
Infection sites, *N* (%)			0.707^*^
Pulmonary infection	29 (82.9%)	54 (85.7%)	
Non-pulmonary infection	6 (17.1%)	9 (14.3%)	
Invasive ventilation, *N* (%)	15 (42.9%)	19 (30.2%)	0.206^*^
Underlying disease			
COPD, *N* (%)	27 (77.1%)	51 (81.0%)	0.654^*^
Pulmonary fibrosis, *N* (%)	5 (14.3%)	6 (9.5%)	0.703^*^
Coronary heart disease, *N* (%)			0.745^*^
Stable	24 (68.6%)	47 (74.6%)	
Coronary ischemia	5 (14.3%)	6 (9.5%)	
Hypertension, *N* (%)	29 (82.9%)	49 (77.8%)	0.550^*^
Atrial fibrillation, *N* (%)	16 (45.7%)	28 (44.4%)	0.904
Diabetes mellitus, *N* (%)	19 (54.3%)	24 (38.1%)	0.122^*^
Chronic kidney disease, *N* (%)	24 (68.6%)	31 (49.2%)	0.064^*^
Neurological disease, *N* (%)	9 (25.7%)	22 (34.9%)	0.348^*^
Malignant tumor, *N* (%)	5 (14.3%)	7 (11.1%)	0.890^*^
Thyroid hypofunction, *N* (%)	1 (2.9%)	4 (6.3%)	0.652^*^
Lactate at baseline, mmol/L, median (IQR)	1.2 (0.9, 1.5)	1.2 (0.9, 1.4)	0.663^#^
Lactate at end-point, mmol/L, median (IQR)	4.6 (3.7, 5.5)	2.1 (1.6, 2.6)	<0.0001^#^
Serum creatinine, mg/dl, median (IQR)	1.57 (0.89, 2.68)	1.12 (0.79, 1.56)	0.022^#^
Albumin, g/L, median (IQR)	31.423 ± 5.3973	32.346 ± 3.9450	0.378^†^
Hemoglobin, g/dl, median (IQR)	8.9 (8.0, 10.1)	10.0 (8.8, 11.8)	0.018^#^
CK, U/L, median (IQR)	29.3 (21, 56)	36 (19, 49.9)	0.982^#^
LDH, U/L, median (IQR)	301 (188, 510)	241 (172, 345)	0.247^#^
ALT, U/L, median (IQR)	10 (6, 23)	16 (10, 30)	0.275^#^
AST, U/L, median (IQR)	29 (16, 81)	26 (18, 41)	0.643^#^
TNI, ng/ml, median (IQR)	0.17 (0.055, 0.304)	0.044 (0.017, 0.107)	<0.0001^#^
D-dimer, μg/ml, median (IQR)	3.14 (1.87, 5.11)	1.71 (1.24, 3.09)	0.003^#^
Arterial blood glucose, mmol/L, median (IQR)	8.7 (6.5,11.1)	6.8 (5.6, 8.5)	0.013^#^
PaO2, x ± s	90.34 ± 9.36	91.95 ± 8.15	0.368^†^
PaCO2, x ± s	39.51 ± 4.16	38.70 ± 4.34	0.397^†^
eGFR, ml/min/1.73 m2, median (IQR)	32.0 (19.0, 56.0)	45.0 (32.0, 63.0)	0.026^#^
SOFA, median (IQR)	10 (9, 15)	6 (5, 9)	<0.0001^#^
30-day mortality, *N* (%)	17 (48.6%)	18 (28.6%)	0.015^*^

*IQR, interquartile range; COPD, chronic obstructive pulmonary disease; CK, creatine kinase; LDH, lactate dehydrogenase; ALT, alanine dehydrogenase; AST, aspartate transaminase; TNI, troponin I; pro-BNP, pro-brain natriuretic peptide; eGFR, estimate glomerular filtration rate; SOFA, sequential organ failure assessment; PaO_2_ partial pressure of oxygen*.

* *Chi-square test*.

# *Mann–Whitney U test*.

† *t-test*.

### Risk Factors Associated With Linezolid-Induced Lactic Acidosis According to Multivariate Binary Logistic Regression

Significant factors (*p* < 0.1) in the univariate analysis were entered into a multivariate binary logistic regression model to determine their independent effects. No associations were observed between LILA and CKD, serum creatinine levels, hemoglobin levels, TNI levels, pro-brain natriuretic peptide levels, D-dimer levels, or eGFR. Duration of linezolid therapy ≥ 9 days (OR, 3.541; 95% CI, 1.161–10.793; *p* = 0.026, Table 4), arterial blood glucose level ≥ 8 mmol/L (OR, 4.548; 95% CI, 1.507–13.725; *p* = 0.007, Table 4), and a high SOFA score (OR, 1.429; 95% CI, 1.213–1.685; *p* < 0.0001, Table 4) were associated with LILA.

**Table 4:** Multivariate analyses of risk factors for linezolid-induced lactic acidosis.

	**OR (95% CI)**	***P*-value**
Duration of linezolid, ≥9 days	3.541 (1.161–10.793)	0.026
Arterial blood glucose, ≥8 mmol/L	4.548 (1.507–13.725)	0.007
SOFA	1.429 (1.213–1.685)	<0.0001

### Establishment of the Risk Prediction Model

The risk of LILA can be predicted by three factors: the duration of linezolid therapy, arterial blood glucose level, and SOFA score.

Logit (*P*) = −5.263 + 1.264 × duration of linezolid (≥9 = 1, <9 = 0) + 1.515 × arterialblood glucose (≥8 = 1, <8 = 0) + 0.357 × SOFA score.

The probability of LILA in each patient: *P* = e^Logit(*P*)^/(1 + e^Logit (*P*)^).

**Figure 3:**
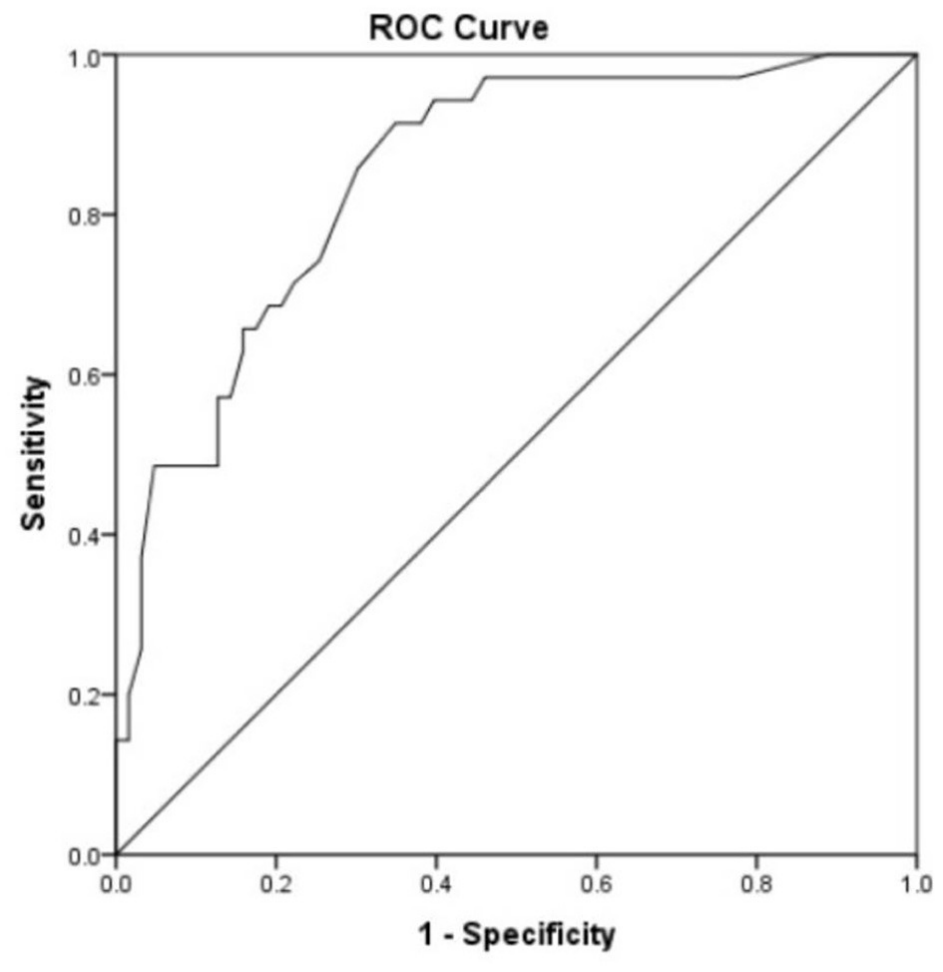
Receiver operating characteristic curve analysis was used to evaluate the accuracy of the risk prediction model. Area under the curve was 0.849 with 95% CI (0.772–0.926) (*p* < 0.0001). Cutoff was 0.2825 with a sensitivity of 91.4%, a specificity of 65.1%, a negative predictive value of 93.2%, and a positive predictive value of 59.3%.

Receiver operating characteristic curve analysis was used to evaluate the accuracy of the risk prediction model. The area under the curve was 0.849 (95% CI 0.772–0.926, *p* < 0.0001, Figure 3). The cutoff value was 0.2825, with a sensitivity of 91.4%, a specificity of 65.1%, a negative predictive value of 93.2%, and a positive predictive value of 59.3%.

### Validation of Model Stability in 32 Additional Patients

To verify the stability of the risk prediction model, 32 patients older than 85 years from the First Medical Centre of Chinese People's Liberation Army General Hospital between January and October 2019 were included. All the patients had blood gas analysis and arterial lactate levels monitored during the use of linezolid. The gold standard for the diagnosis of LILA was the combination of blood gas analysis and arterial lactate measurements.

According to the four-fold table (Table 5), the sensitivity of the risk prediction model was 100%, the specificity was 80%, the negative predictive value was 100%, and the positive predictive value was 58.3%.

**Table 5:** Thirty-two patients older than 85 years verified the stability of the risk prediction model.

**Risk prediction mode**	**Gold standard**		**Total**
	**Lactate acidosis**	**Non-lactate acidosis**	
Lactate acidosis	7	5	12
Non-lactate acidosis	0	20	20
Total	7	25	32

## Discussion

Linezolid has been used as a powerful medicine to treat multidrug-resistant gram-positive bacterial infections; however, this type of antibiotic could cause type B lactic acidosis due to inhibition of mitochondrial oxidative phosphorylation in the absence of apparent tissue hypoxia. LILA in the elderly has not been deeply concerned for a long time until a notable incidence of LILA was reported. LILA was supposed to affect the survival of patients in clinical practice.

In the current study, 35.7% of very elderly patients (older than 85 years) receiving linezolid therapy were observed with lactic acidosis. This overall percentage of LILA was slightly higher than previously reported, 6.8% in Im's study and 10.6% in Mori's study in all age groups (17–20). The reason for the higher percentage of LILA could be partly caused by the patients with mild disease who were excluded from the study due to the absence of blood gas monitoring. The very elderly population included in our study had relatively severe disease and multiple diseases or called comorbidities. In previous studies, those patients whose lactate levels were not being monitored were included in the non-lactic acidosis group, which might also lead to the underestimation of LILA incidence (20).

In our research, we found that the 30-day mortality rate was 48.6% in the lactic acidosis group, which was significantly higher than that in the control group (*p* < 0.05). In a systematic review and meta-analysis of 47 cases of LILA retrieved from PubMed, 25.5% of the patients died, indicating a high risk of mortality associated with LILA (21).

Previous reports have shown that LILA is associated with a longer duration of medicine therapy (15, 16, 22–24). In a retrospective study, duration of linezolid therapy > 6 weeks was a risk factor for LILA (19), but LILA has been reported to occur after a shorter duration of linezolid therapy (4 h−7 days) (21, 25–28) and even earlier in children with a median time of 2 (1, 13) days (29). Del Pozo had shown that the median duration of the administration of linezolid in patients with lactic acidosis was 8 days (20). We found that the duration of linezolid therapy ≥ 9 days was a risk factor for LILA in the very old population, suggesting that LILA in the very elderly population could occur after a short course of medication, although the underlying mechanism has not yet been clarified. Therefore, early and routine monitoring of lactate levels and blood gases is necessary for geriatric wards.

We also discovered that when the arterial blood glucose level was ≥8 mmol/L, the risk of lactic acidosis was elevated. In the absence of oxygen or mitochondrial oxygen use disorders, glucose produces lactic acid through anaerobic fermentation glycolysis. This could be the major cause of why lactic acidosis is prone to occur with elevated blood glucose.

A high SOFA score could also be a risk factor for LILA, and the risk of lactic acidosis increased 0.429 times for every one-point increase in the SOFA score, suggesting that very elderly patients with high SOFA scores are more prone to lactic acidosis. One study of 10 cases of LILA found that a SOFA score ≥ 11 and the duration of linezolid therapy ≥ 7 days were not risk factors for LILA (20). This was inconsistent with our results, which may be related to the univariate analysis used in previous studies.

Renal dysfunction might affect the excretion of linezolid, leading to the increased blood concentration of linezolid, and thereby induced LILA. Del Pozo (20) found that an eGFR ≤ 30 ml/min (OR, 7.4; 95% CI, 1.0–84.4, *p* = 0.02) was a risk factor for LILA; however, we did not find that the eGFR was associated with LILA. Actually, the non-renal clearance rate of linezolid was 65%. Furthermore, 30% of lactic acid clearance occurs in the kidney. Unless the lactic acid level is above 6–10 mmol/L, it will not start to be excreted by the kidneys (1). Therefore, eGFR may only be associated with severe hyperlacticacidemia. In our study, lactate levels were mostly mildly to moderately elevated; no correlation was confirmed between LILA and eGFR.

LILA may be associated with linezolid overexposure. In a case report, patients who received regular doses of linezolid (600 mg twice daily) with significant plasma overexposure to linezolid (minimum concentration, 26.99 mg/L) developed significant lactic acidosis (26). A recent study showed that patients ≥ 80 years had concentrations three times higher compared with patients <40 years, suggesting a positive correlation between linezolid concentrations and patient age (30). This may be why the high incidence and early onset of LILA in elderly patients in our study. More data are needed to illustrate the correlation between the concentration of linezolid and LILA.

Based on the multivariate logistic regression analyses, we established a risk prediction model for the occurrence of LILA with high sensitivity and specificity (91.4 and 65.1%, respectively), with a cutoff value of 0.2825. Verification of the model in 32 patients from another medical center showed that it was very stable. Therefore, the risk prediction model can be applied to the very elderly population.

The mechanism underlying LILA is still unclear. Human cells only contain L-lactate dehydrogenase that exclusively synthesizes L-lactic acid. Some colonic carbohydrate-fermenting bacteria produce D-lactic acid by D-lactate dehydrogenase. The usual lactic acid laboratory tests cannot detect D-lactic acid. Therefore, linezolid might induce the production of L-lactic acid by human cells. Linezolid inhibits 23S ribosomal RNA (rRNA) from the 50S subunit of the bacterial ribosome, similar to human mitochondrial 16S rRNA. Hence, linezolid might produce toxic mitochondrial effects by binding to human mitochondrial 16S rRNA and inhibiting mitochondrial protein synthesis (24). Human mitochondrial DNA polymorphisms (A2706G and G3010A) have been found associated with LILA (31, 32), although this finding remains controversial due to the high frequency (up to 80%) of the polymorphisms and the relatively rare occurrence of LILA (33).

To the best of our knowledge, this is the first study to analyze the occurrence of LILA in a large sample of patients older than 85 years. Moreover, we established a risk prediction model to predict the occurrence of LILA. There are some limitations to this study. First, as a retrospective study, the concentration of linezolid was not monitored. A recent preliminary result by our group showed that the trough concentrations of linezolid in elderly patients were more than 8 mg/L. Further studies are of great interest soon. Second, the included patients of very old age presenting relatively severe illness might contribute to the mortality. Third, the sample size was relatively small, and other adverse effects of linezolid such as thrombocytopenia, erythrocytopenia, and nervous lesion were worth further investigation. Furthermore, the mechanism underlying LILA needs to be further studied.

## Conclusion

This study identified the risk factors for LILA and established a stable risk prediction model. LILA can occur in very elderly patients after a relatively shorter duration of linezolid, indicating that the close monitoring of blood gases and arterial lactate levels during the administration of linezolid is necessary.

## Data Availability Statement

The original contributions presented in the study are included in the article/Supplementary Material, further inquiries can be directed to the corresponding author/s.

## Ethics Statement

The studies involving human participants were reviewed and approved by the ethics committee of Chinese People's Liberation Army (PLA) General Hospital (Ethical approval number: S2018-162-01). Written informed consent for participation was not required for this study in accordance with the national legislation and the institutional requirements.

## Author Contributions

HL, GX, and TL contributed to the study design. JWa and YQ contributed to the collection of clinical data. HL, XF, and ML contributed to the data analysis. TL, CH, and JWu drafted the article. All authors contributed to the article and approved the submitted version.

## Conflict of Interest

The authors declare that the research was conducted in the absence of any commercial or financial relationships that could be construed as a potential conflict of interest.
