# Supplementary material for: Incidence and Associated Risk Factors for Lactic Acidosis Induced by Linezolid Therapy in a Case–Control Study in Patients Older Than 85 Years
Source: Front Med (Lausanne). 2021 Feb 25;8:604680. doi: 10.3389/fmed.2021.604680 (PMC7959744; doi:10.3389/fmed.2021.604680)
Supplement: Supplementary file 1 [file Table_1.DOCX]

Supplementary Material

**Supplementary TABLE.** Patient characteristics and clinical factors

|  | | | **Teicoplanin**  **(n=199)** | **Linezolid**  **(n =108)** | ***P* value** |
| --- | --- | --- | --- | --- | --- |
| Male sex, N (%) | | | 171(85.9%) | 99(91.7%) | 0.140* |
| Age, years, median (IQR) | | | 94[92,97] | 94.5[91,97] | 0.677^#^ |
| Duration of antibiotics, days, median (IQR) | | | 9[6,13] | 9[6,12] | 0.527^#^ |
| Infection site, N (%) | | |  |  | 0.006* |
|  | Pulmonary infection | | 144(72.4%) | 93(86.1%) |  |
|  | Non-pulmonary infection | | 55(27.6%) | 15(13.9%) |  |
| Invasive ventilation, N (%) | | | 78(39.2%) | 34(31.5%) | 0.180* |
| Underlying disease | | |  |  |  |
|  | COPD, N (%) | | 149(74.9%) | 85(78.7%) | 0.452* |
|  | Pulmonary fibrosis, N (%) | | 26(13.1%) | 12(11.1%) | 0.620* |
|  | Coronary heart disease, N (%) | |  |  | 0.009* |
|  |  | Stable | 181(91.0%) | 87(80.6%) |  |
|  |  | Coronary ischemia | 18(9.0%) | 21(19.4%) |  |
|  | Hypertension, N (%) | | 157(78.9%) | 86(79.6%) | 0.880* |
|  | Atrial fibrillation, N (%) | | 120(60.3%) | 51(47.2%) | 0.028* |
|  | Diabetes mellitus, N (%) | | 83(41.7%) | 52(48.1%) | 0.278* |
|  | Chronic kidney disease, N (%) | | 106(53.3%) | 55(50.9%) | 0.695* |
|  | Neurological disease, N (%) | | 32(16.1%) | 33(30.6%) | 0.003* |
|  | Malignant tumor, N (%) | | 14(7.0%) | 14(13.0%) | 0.085* |
|  | Thyroid hypofunction, N (%) | | 4(2.0%) | 6(5.6%) | 0.182* |
| Serum creatinine, mg/dl, median (IQR) | | | 101.0[75.0,143.0] | 120.5[74.5,197.5] | 0.081^#^ |
| SOFA, median (IQR) | | | 8[5,11] | 9[6,13] | 0.013^#^ |

*IQR, interquartile range; COPD, Chronic obstructive pulmonary disease; SOFA, Sequential organ failure assessment*

*^*^Chi-square test*

*^#^Mann-Whitney U test*
